# Effectiveness of a peer-based intervention on loneliness and social isolation of older Chinese immigrants in Canada: a randomized controlled trial

**DOI:** 10.1186/s12877-020-01756-9

**Published:** 2020-09-21

**Authors:** Daniel W. L. Lai, Jia Li, Xiaoting Ou, Celia Y. P. Li

**Affiliations:** 1 Faculty of Social Sciences, Hong Kong Baptist University, Hong Kong, China; 2 Department of Applied Social Sciences, The Hong Kong Polytechnic University, Hong Kong, China; 3 The Calgary Chinese Elderly Citizens’ Association, Calgary, Canada

**Keywords:** Chinese aging immigrants, Peer-based intervention, Social isolation, Social participation, Loneliness, Randomized controlled trials

## Abstract

**Background:**

Social isolation is a key concern for immigrant older adults. We examined the effectiveness of a peer-based intervention in reducing loneliness, social isolation, and improving psychosocial well-being with a sample of aging Chinese immigrants.

**Methods:**

Sixty community-dwelling older Chinese immigrants aged 65 and older were randomly assigned to an intervention group and a control group (*n* = 30 each) in a randomized control parallel trial design. Intervention group participants received an eight-week peer support intervention. Twenty-four volunteers aged 48 to 76 engaged in two-on-one peer support through home visits and telephone calls to provide emotional support, problem-solving support, and community resource sharing. Social workers who are not blinded to the group assignment measured the changes of both the intervention group and the control group participants in a range of psychosocial outcomes including three primary outcomes (loneliness, social support, barriers to social participation) and five secondary outcomes (depressive symptoms, anxiety, life satisfaction, happiness, and purpose in life).

**Results:**

The 30 intervention group participants showed a statistically significant decrease in loneliness and increase in resilience when compared to the 30 control group participants. They reported fewer barriers to social participation, fewer depressive symptoms, increased life satisfaction, and happiness while no such improvements were observed in the control group.

**Conclusions:**

There is a need to further examine the use of peer-based interventions for both program effectiveness and delivery efficiency. In the era of population aging and increasing immigration, diverse aging adults can be trained to fill volunteer support roles via peer-based intervention approaches.

**Trial registration:**

ISRCTN, ISRCTN14572069, Registered 23 December 2019 – Retrospectively registered.

## Background

The global trends of aging and international migration have resulted in the changes in the age and ethnic composition of population, particularly in countries with a strong history of being open to immigration such as Canada, the United States, the United Kingdom, and Australia [1]. The global increase of older immigrants is a fact. For instance between 1990 and 2013, this population has increased for over 42% from 26 million to 37 million worldwide [2].

Social isolation and loneliness have detrimental effects on older people’s physical and mental health [3, 4]. Older immigrants are vulnerable to social isolation and loneliness due to language barriers, cultural differences, discrimination, and evolving familial caregiving dynamics. For instance, immigrant Canadians experience greater loneliness than native-born Canadians [5] and are vulnerable to psychosocial challenges such as emotional distress, depression, and anxiety [6]. These Canadian findings are consistent with ones in other countries in which challenges related to adaptation, access to services, lack of community participation, language and cultural barriers, and sense of exclusion were reported in older immigrants. For instance, a recent American study pointed out that in addition to language barriers, cultural beliefs and family shame may also prevent older immigrants from using health services, especially mental health services [7]. In New Zealand, studies have reported that older immigrants are less active in the community, less likely to engage with health services, and more likely feel excluded by the community due to language barriers and cultural difference [8–10]. In Australia, research shows that many older immigrants may be unwilling to access health care services due to limited English proficiency and cultural beliefs and knowledge [11]. Review papers in Canada [12], the United States [13], and New Zealand [14] all have pointed to similar challenges faced by older immigrants in terms of access barriers related to language, cultural values and beliefs, inequitable resources, access barriers, and various structural challenges. In the United Kingdom, research has indicated that social care services have usually left the older immigrants out [15].

### Conceptual background and evidence of peer-based interventions

Dynamic social impact theory emphasizes the importance of background similarity in peer support relationships, suggesting that communication between people with a similar cultural background can be more effective and lead to behaviour changes than in a cross-cultural context [16]. Therefore, it is hypothesized that comparing to interventions performed by others such as professionals, peer-based interventions are advantageous and can generate better outcomes. Regarding the mechanisms in which peer-based intervention can influence people’s health, social cognitive theory suggested that people’s health-related beliefs, values and behaviors can be changed to be more like peers due to the urge of conformity [16]. Additionally, social support theory suggests that emotional, instrumental, or informational support from peers can help people to overcome difficulties and therefore avoid adverse health outcomes [17].

Support for peer is usually referred to support with knowledge, help, and experience by peers, which could be family members, friends, or people from the same neighbourhood [18, 19]. In peer-based interventions, participants build effective working alliances and trust with supporters or mentors and reveal their problems and feelings with their peers. Theoretical perspectives provide explanations on how peer-based interventions can promote health-related behavioural changes and health outcomes.

Previous empirical studies have shown the effectiveness of peer-based interventions in reducing loneliness among socially isolated older adults. For instance, one study found that widowed seniors tended to experience lower emotional loneliness and social isolation after attending peer-based support groups [20]. A British study of a peer-based telephone support program showed a reduction in participants’ experiences of social isolation and loneliness after several rounds of talking with peer supporters [21]. Peer-based interventions also contribute to reduced depression and helplessness, better health management, increased self-efficacy, coping skills, self-care activities, self-confidence, and health-related behaviours such as physical activity and reduced smoking in aging populations [18, 22]. A small number of studies identified the negative impacts of peer-based interventions and they were mostly linked to lack of peer training, which suggested the importance of structured training given by the professionals to the peers [23, 24]. Peer-intervention programs are also beneficial to peer mentors. By increasing their knowledge, expanding social networks, and watching changes among their mentees, peers experience positive feelings and increased confidence [25].

### The current intervention study

In Canada, Chinese older immigrants represent one of the largest immigrant and ethnic minority groups who are non-white and non-Caucasian [26]. Compared to older immigrants in other ethnic minority groups, such as Southeast Asians, Chinese older immigrants tend to have lower levels of acculturation and lower English proficiency, and may therefore face more difficulties in integration into mainstream society and greater vulnerability to social isolation and loneliness, which deserves attention of practitioners [5]. The Mental Health Commission of Canada has highlighted the need for peer-based support programs to support older immigrants [27].

This study involved the implementation and assessment of a peer-based intervention program – the Chinese Community Helpers Program (CCHP) – in Calgary, Canada. The intervention aimed to increase social support and positive ties among older Chinese immigrants, especially those at greater risk of social isolation (such as those living alone or who are widowed), bridge formal and informal support, and strengthen helping skills and knowledge of community helpers in terms of mental health and aging. This study aimed to examine the effectiveness of the peer-based intervention on older Chinese immigrants’ psychosocial well-being. The primary expected outcomes of the CCHP include reduced loneliness, increased social support, and reduced barriers to social participation of participants who received peer-based interventions. Secondary outcomes include other forms of psychological well-being, including reduced depressive symptoms and anxiety and increased happiness, life satisfaction, resilience, and purpose in life. The study addressed the following questions: (1) can CCHP reduce the loneliness, increase social support, and reduce barriers to social participation of older Chinese immigrants in Calgary, Canada? (2) can CCHP reduce depression and anxiety, increase happiness, life satisfaction, purpose in life, and resilience of older Chinese immigrants in Calgary, Canada?

## Methods

### Study design and participants recruitment

This study used a randomized controlled trial design. We used the primary outcome loneliness to calculate the sample size needed. We adopted the effect size of 0.6 from a similar peer-based trial conducted among socially isolated Hong Kong Chinese older adults to reduce their loneliness [28]. With a power of 0.8 (alpha = 0.05, two-sided), 26 participants for each group and 52 in total were needed to detect such an effect size. G*Power 3.1 was used to calculate the sample size [29]. Taking the potential dropout into consideration, we aimed for 30 participants for each group and 60 in total for the baseline. Intervention group participants received an eight-week peer-based intervention.

Sixty participants were recruited by promoting information about the CCHP in Chinese immigrant communities and independent living and assisted living facilities for Chinese older people in the community. Professional practitioners of the host organization also promoted the program through local Chinese radio programs in Calgary, Canada, word of mouth, and putting program notices in the weekly organization activity newsletters that were printed in free local Chinese newspapers. The recruitment of older participants start from 15 of June 2018. Inclusion criteria for participants were: 1) aged 65 and older, 2) independent in activities of daily living, 3) self-reported as socially isolated or in need of social support, and 4) not clinically diagnosed with a mental disorder or cognitive impairment.

Social workers would screen the eligibility of participants when they signed up. In addition to the above four criteria, those who were evaluated by the social workers as in urgent needs of immediate professional help were excluded from this peer-based intervention and referred to professional help. Such participants may include those who was undergoing life transition or under critical situation such as loss of loved one, mental health challenges, or severe family problem (e.g. abusive relationship), and severe depressive mood.

Sixty-six participants signed up for the program, and six were excluded due to severe needs that required professional support unable to be fulfilled by peer volunteers. After the screening, eligible participants were randomly assigned to either an intervention group (*n* = 30) or a control group (*n* = 30). All of the participants were blinded to the group assignment. The name of the participants were put on a participant’s list in sequence once they signed up for the programs. They would be assigned randomly based on the number they were in, for example, participants with an odd number go to control group, and those with an even number go to intervention group. The social workers were assigned to intake the participants with sequence with one participant interval between control and intervention group. However, three participants were considered by the social workers as in urgent need due to being in social isolation were given priority to be assigned into intervention group as special cases due to ethical concerns.

### Peer training and intervention implementation

Twenty-four Chinese immigrant volunteers aged 48 to 76 years old were recruited and trained between January and April 2018. All the volunteers received 15 h of training by the fourth author who is a professionally trained Registered Social Worker with over two decades of experience in working with older people and mental health issues. The training contents focused on topics such as basic understanding of mental health, knowledge and skills to offer peer support, how to deal with grief and loss, self-help skills, stress management, goal setting, and how to build healthy relationships. Five classes of core volunteer training were held on 21st of January, 23rd and 30th of April, and 13rd and 20th of August in 2018.

The intervention was from mid July to end of December 2018. In the first week, social workers connected each intervention group participant with two peer supporters. The social workers were not blinded to the group assignment and assigned the trained volunteers to visit participants in the intervention group. Over subsequent weeks, each intervention group participant received two-on-one peer support services through home visits, telephone calls, and activities such as emotional support, referrals, help to establish goals such as self-care and social engagement, problem solving, and mental health and community resources. The reason for a two-on-one match was due to the standard professional practice safety protocol preventing sending only one staff members or volunteer for home visit. Having two peer supporters would also have the benefit of allowing them to be able to pay additional attention to both the individual and living environment in the home visit and subsequent support intervention process. Although the volunteers were unable to provide professional intervention or therapy, they actively listened to and respected the needs of the older participants. During the matching process, there were considerations made regarding the gender, age, language, and education background so that some form of commonalities were linked between the older person and the peer-supporters. Peer supporters also shared personal experiences with participants. Intervention group participants were invited to attend two monthly peer support group meetings organized by a trained staff program coordinator with professional training in social work and a Registered Social Worker, where they met with other participants and peer supporters, intended to help them develop strong, supportive, and sustainable social connections with other older people.

Control group participants only received brief telephone calls from the program coordinator over an eight-week period. The project team believes that these regular calls would just be used for normal social greetings or answering any questions related to program information raised by the call receivers. While a sense of care may be felt by the call receivers, which is common in all forms of social interactions that the call receivers experienced in their routine daily interactions with others, the calls would not explicitly extend any invitation to actions that would trigger further engagement in social participation in programs or social activities. In situation when the participants in the control group were to display emerging health or emotional crisis, the program coordinator would initiate the necessarily crisis intervention over the phone or invite the participant to attend professional support sessions afterward. Under this circumstance, the participant would be excluded from the control group of the study.

### Data collection

**Fig. 1:**
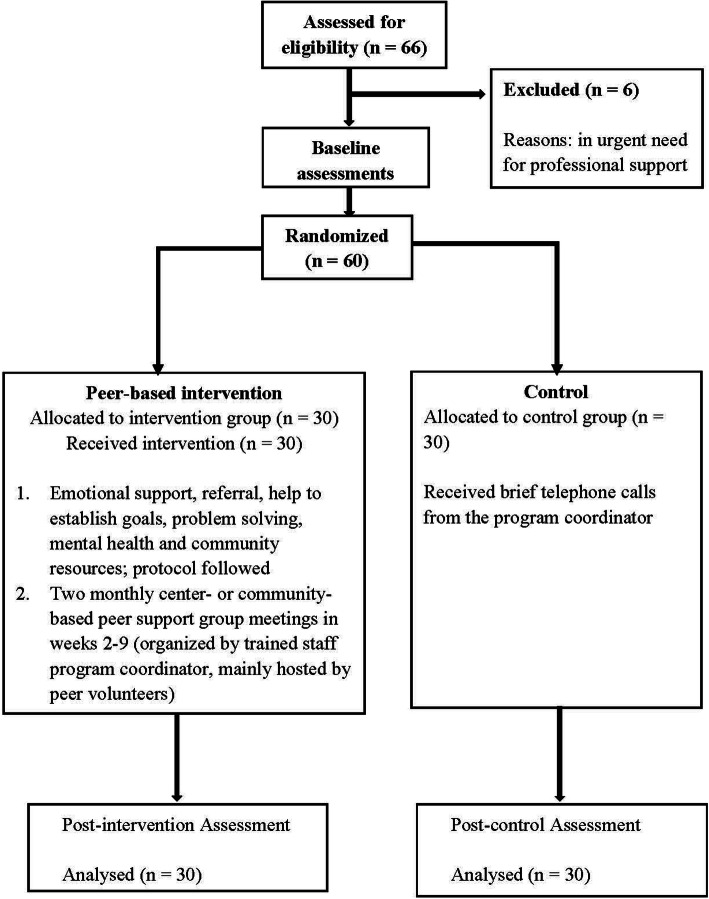
The intervention and evaluation process

Baseline and post-tests with control and intervention groups were conducted by social workers via individual interviews took place during home visit to each of the participants in week 1 and 10, respectively. The process of the study design and the procedures is shown in Fig. 1. Ethics approval was granted by the first author’s university. Written consent was obtained from all participants. The control group will be provided the same peer-based intervention after the project was completed and positive outcomes were found.

### Measures

#### Primary outcomes

Loneliness was measured using the De Jong Loneliness Scale-6, which has been translated into Chinese and validated among Hong Kong Chinese older adults with satisfactory reliability and validity [30]. The first three questions measure emotional loneliness (e.g. ‘*I experience a general sense of emptiness*’). Answers ‘yes’ or ‘more or less’ were coded as 1 and ‘no’ was recoded as 0. The other three questions measure social loneliness (e.g. ‘*There are plenty of people I can rely on when I have problems*’). Answers ‘more or less’ or ‘no’ were recoded to 1, and ‘yes’ was recoded as 0. Overall scores range from 0 to 6, with 0 indicating no loneliness and a higher score indicating more severe loneliness [30].

Social support was measured using the Lubben Social Network scale (LSNS) [31], a 10-item scale that has been translated into Chinese and validated among Hong Kong Chinese older adults. The scale measures family networks (3 items), friend networks (3 items), and interdependent relationship (4 items). Higher overall scores indicate higher levels of social support. Barriers to social participation was measured using the Keele Assessment of Participation [32], which was translated and back-translated for this study. This 15-item scale measures an individual’s restrictions to participation in activities such as daily living, work, and social activities, and includes four screening questions (‘*If yes, proceed to the next question’*). Answers include ‘all the time’, ‘most of the time’, ‘some of the time’, ‘a little of the time’, and ‘none of the time’. The first two were recoded as 0, and the other three recoded as 1. Overall scores range from 0 to 11, with 0 indicating no restriction in social participation. The scale demonstrated an acceptable level of internal consistency within this sample with the Cronbach’s alpha being 0.64 and 0.65 for the baseline and post-intervention evaluation, respectively.

#### Secondary outcomes

Life satisfaction was measured by a single question (‘*In general, how satisfied are you with your life*?’), with answers ranging from 1 = very unsatisfied to 4 = very satisfied. The score was further recoded into two categories: unsatisfied (answering ‘very unsatisfied’ and ‘unsatisfied’) and satisfied (answering ‘satisfied’ and ‘very satisfied’). Happiness was also measured by a single-item question (‘*In general, how happy do you feel?*’), from 0 = very unhappy to 10 = very happy. We chose the two single-item measurements because these two single-item questions have been widely used in research with Chinese older adults [33, 34]. Moreover, simple structured assessment tools were needed due to the characteristics of the older participants who may unable to endure prolong interviews and complicated interview protocols.

Depression was measured using the General Depression Scale (GDS-4), which has been translated in Chinese and validated among Hong Kong Chinese older adults [35, 36]. It includes four questions (e.g. *‘Are you basically satisfied with your life*?’, ‘*Do you feel that your life is empty*?’), with participants answering yes = 1 or no = 0. Answers to positive items were recoded, with overall scores ranging from 0 to 4 and higher overall scores indicating more severe depressive symptoms. A cut-off point of 2 was adopted, as suggested by Cheng and Chan [36]. Overall scores of 0 and 1 were coded as ‘no depressive symptom’ and scores of 2 and greater were coded as ‘depressive’. Anxiety was measured using the Chinese version of the Geriatric Anxiety Inventory – Short Form (GAI-SF) [37, 38]. It includes five statements (e.g. ‘*I worry a lot of the time*’), with participants answering yes = 1 or no = 0. Overall scores range from 0 to 5, with higher scores indicating greater anxiety.

Resilience was measured using the two-item Connor-Davidson Resilience Scale (CD-RISC 2) [39], which includes two statements measuring whether an individual is ‘*able to adapt to change*’ and ‘*tends to bounce back after illness or hardship*’. The Chinese version has been validated [40]. Answers range from 0 = not true at all to 4 = true nearly all the time. Overall scores range from 0 to 8, with higher scores indicating greater resilience. Purpose in life was measured using the seven-item subscale of Ryff’s Psychological Well-being Scale (e.g. ‘*I tend to focus on the present, because the future nearly always brings me problems*’;) [41]. A Chinese version has been translated [42]. Answers range from 1 = completely disagree to 6 = completely agree. Answers to negative items were recoded. Higher overall scores indicate a higher level of purpose in life.

### Statistical analysis

The intent-to-treat (ITT) approach was adopted for all data analyses. Independent samples t-tests (or Mann Whitney’s test for non-normal distributed continuous variables) were used to test baseline differences between intervention and control groups. Skewness and Kurtosis normality test was conducted. Chi-square tests were conducted for categorical variables, and Fisher’s exact test were conducted if the size of any category was small (< 5) [43]. Analysis of variance was used to evaluate continuous outcomes, with time being the within-group factor and control/intervention as the between-groups measure. Factorial logistic regressions were conducted for depression and life satisfaction, which are dummy variables. Conclusion about the effectiveness of the intervention were based on the time by group interaction coefficients of the models.

Paired samples t-tests (or Wilcoxon signed rank test for non-normal distributed continuous variables) were conducted to compare pre- and post- scores for all outcome variables for the intervention and control groups respectively. The magnitude of changes between the intervention and control groups was compared via independent samples t-test (or Mann Whitney’s test for non-normal distributed continuous variables). Cohen’s d was calculated for the between-group differences variables that showed a significant change. A two-tail *p*-value < 0.05 was set as the significant confidence level. All analyses were performed using Stata 15.0 (StataCorp).

## Results

### Sample characteristics

The final sample included 30 participants in the experimental group and 30 in the control group. There were no dropout and all of the participants finished both the baseline and post-evaluation. Participants’ socio-demographic characteristics are shown in Table 1. Most participants were aged over 80 and most were female. Nearly all participants were Chinese, with only one from a Southeast Asian country. Most were born in Mainland China, followed by Hong Kong, Macau, and Vietnam. Chinese, including Mandarin, Cantonese, and other dialects, was their mostly frequently used language. Almost half the participants were widowed. Over half reported either often or sometimes having difficulties in performing daily activities and had reduced their activities due to a physical or mental health problem. There were no significant differences in socio-demographic characteristics between intervention and control groups, with the exception of age distribution, where intervention group participants tended to have an older age than control group participants at a marginally significant level (*p* = 0.051).

**Table 1:** Sample characteristics of the intervention group (*n* = 30) and control group (*n* = 30)

Variables	Intervention *n* = 30		Control *n* = 30		Chi-square (*p*-value)
	%	n	%	n	
Gender					.287(.592)
Female	66.7	20	60	18	
Male	33.3	10	40	12	
Age					40.000(.051)
61–70	20	6	13.3	4	
71–80	10	3	43.3	13	
81–90	36.7	11	30	9	
91 and older	33.3	10	13.3	4	
Language most often spoken at home					1.111(.574)
English	0	0	3.3	1	
Chinese unspecified	83.3	25	83.3	25	
Mandarin	16.7	5	13.3	4	
Country of birth					2.864(.581)
Mainland China	70	21	80	24	
Hong Kong	16.7	5	10	3	
Macau	3.3	1	0	0	
Vietnam	10	3	10	3	
Number of years in Canada					37.312(.201)
0–10	0	0	6.7	2	
11–20	26.7	8	10	3	
21–30	26.7	8	40	12	
31–40	20	6	13.3	4	
41 and above	16.7	5	13.3	4	
Cannot remember	10	3	16.7	5	
Marital status					1.758(.624)
Married	36.7	11	50	15	
Widowed	50	15	43.3	13	
Separated	3.3	1	3.3	1	
Divorced	10	3	3.3	1	
Difficulty of performing activities					1.864(.394)
Yes, sometimes	30	9	36.7	11	
Yes, often	43.3	13	26.7	8	
No	26.7	8	36.7	11	
Physical/mental condition or health problem reduces activities					.821(.663)
Yes, sometimes	46.7	14	46.7	14	
Yes, often	13.3	4	6.7	2	
No	40	12	46.7	14	

Table 2 shows the outcome comparison of the intervention and control groups at baseline. There was a significant difference between the two groups in terms of barriers to social participation (*p* = 0.003). Intervention group participants (M = 5.33, SD = 2.22) had a significantly higher level of barriers to social participation than those in the control group (M = 3.8, SD = 1.064).

**Table 2:** Comparison of outcome measures between intervention and control groups at baseline

Outcomes	Intervention (*n* = 30)	Control (*n* = 30)	Difference (*p*-value)
	M (SD) / n (%)	M (SD) / n (%)	
**Primary outcomes**			
Loneliness	3.27 (1.41)	2.63 (1.21)	−1.86(.07)^a^
Social support	22.10 (7.67)	23 (6.64)	.49(.63)^a^
Barriers to social participation	5.333 (2.218)	3.8 (1.064)	−2.939(**.0033**)^b^
**Secondary outcomes**			
Life satisfaction			.052^c^
Unsatisfied	7 (23.33%)	1 (3.33%)	
Satisfied	23 (76.67%)	29 (96.67%)	
Happiness	6.60 (2.03)	6.90 (1.79)	.523(.601)^b^
Depression			.552^c^
No	21 (70%)	24 (80%)	
Yes	9 (30%)	6 (20%)	
Anxiety	.87 (1.59)	1 (1.62)	.296(.767)^b^
Resilience	5.37 (1.52)	5.80 (1.45)	1.131(.263)^a^
Purpose in life	19.33 (9.84)	20.83 (9.63)	.60(.55)^a^

^a^Independent sample t-test; ^b^Wilcoxon rank-sum test; ^c^Fisher’s exact test

### Effectiveness of the intervention

As presented in Table 3, the time by treatment effects in the ANOVA were significant for loneliness, resilience, and barriers to social participation. According to the comparison between the magnitudes of changes for intervention and comparison groups, intervention group participants reported a mean decrease of 1.17 units in loneliness (95% CI, 0.45 to 1.89), a mean decrease of 0.57 units in barriers to social participation (95% CI, 0.13 to 1.00), and a mean increase of 1.37 units in resilience (95% CI, 0.70 to 2.03). The computed Cohen’s d indicated that the effect sizes were large (0.834, 1.051, and 0.908). Intervention group participants also experienced significantly increased levels of happiness, a smaller percentage of participants reporting depressive symptoms, and a higher percentage of participants reporting life satisfaction. These improvements were not observed in the control group.

**Table 3:** Within-group changes before and after the intervention and between-group differences in changes

Outcomes	Intervention (*n* = 30)		Control (*n* = 30)		Time* Group effect: *p*-value	Between-group difference (95% CI)	Effect size (Cohen’s d)
	T2-T1, M (SD)/n (%)	Change (*p*-value)	T2-T1, M (SD)	Change (*p*-value)			
**Primary outcomes**							
Loneliness	−.63 (1.45)	2.39(**.02**)^e^	.53 (1.33)	−2.19(.04) ^e^	**.018**	1.17(.45 to 1.89)	**.834**
Barriers to social participation	−1.27 (2.05)	3.28(**.001**)^d^	.13(.73)	−1.21(.226)^d^	**.018**	−.57(− 1.00 to −.13)	**.908**
Social support	.43 (4.62)	−.51(.61)^e^	−1.4 (4.78)	1.60(.12)^e^	.460	–	–
**Secondary outcomes**							
Life satisfaction	5 (16.7%)	5.00(**.025**)^c^	1 (3.33%)	1.00(.32)^c^	N/A^f^	–	–
Happiness	1.07 (1.53)	−3.36(**.0008**)^d^	.47 (2.00)	−1.11(.27)^d^	.36	–	–
Depression	7 (23.3%)	7.00(**.0082**)^c^	−1 (3.33%)	.20(.65)^c^	.06	–	–
Anxiety	−.5 (1.43)	1.187(.235)^d^	−.27(.91)	1.39(.17)^d^	.657	–	–
Resilience	.73 (1.44)	−2.44 (**.01**)^d^	−.63 (1.13)	2.74(**.006**)^d^	**.004**	−1.37(− 2.03 to −.70)	**1.051**
Purpose in life	−1.6 (4.94)	1.946(.052)^d^	−.67 (6.24)	.76(.445)^d^	.801	–	–

^a^Independent sample t-test; ^b^Wilcoxon rank-sum test; ^c^McNemar’s test; ^d^Wilcoxon signed rank test; ^e^Paired-sample test; ^f^As the expected frequency in table cells was low, statistical analysis could not be performed

### Adjustment analysis

Due to the significant difference in social participation between intervention and control group participants, there is a potential risk of ‘regression to the mean’ effect: if the score in the first measurement is unusually far from the average, it tends to be closer to the average in the following measurements. In this study, barriers to social participation reported by the intervention group were much higher than for the control group. Therefore, changes after the intervention may be due to chance rather than treatment [44]. To test this, we adopted the method recommended by Twisk et al. [45] to use longitudinal analysis of covariance to control for the differences in baseline (Y_1_ = *β*_*0*_+ *β*_*1*_X+ *β*_*2*_Y_0_, where, *Y*_*1*_ = the outcome measured at post-test, *X* = treatment variable, *β*_*1*_ = overall treatment effect and *Y*_*0*_ = outcome variable measured at baseline). After controlling for baseline differences, the overall treatment effect (*β*_*1*_) was no longer significant, which suggests a potential ‘regression to the mean’ effect. It implied that the post-intervention decrease of barriers to social participation among the intervention group may not be due to the intervention, but a random chance because the baseline scores were extremely higher than the mean. There was also a marginally significant difference in two groups’ baseline level of loneliness (*p* = 0.07). Therefore, we adopted the same approach to adjust the baseline level of loneliness. The treatment effect remained after controlling for the baseline differences (*p* = 0.013).

While loneliness and resilience remain the two outcomes with significant treatment effects, we also conducted sensitivity analysis for the two variables to control for the age differences between two groups. The treatment effects remained for both two outcomes with significant *p* values (0.018 and 0.004).

## Discussion

Social isolation and loneliness can cause physical or mental challenges and affecting quality of life for older people [3]. This randomized controlled trial found that a peer-based intervention was effective in reducing loneliness and improving resilience among socially isolated older adults, specifically older ethnic minority immigrants. This program was found to be potentially effective in reducing depressive symptoms, barriers to social participation, and increasing life satisfaction and happiness.

Older immigrants are vulnerable to social isolation due to individual and social factors such as language barriers, lack of social support, lack of knowledge of services, and racism. Peer-based interventions may be particularly effective for this population, who may encounter particular difficulties in accessing formal supports and may tend to underutilize such services when compared to native-born peers [46, 47]. Not only do they encounter difficulties in accessing formal services, but studies also report the diminishing roles of adult children (as a form of informal support) in Chinese immigrant families [48]. In this case, social networks beyond families, namely in co-ethnic communities, become particularly salient in older Chinese immigrants’ daily lives.

The current study findings align with previous research showing that peer-based interventions are an effective approach for reducing loneliness among older people [21, 49]. Different types of interventions can reduce older adults’ loneliness by improving their social skills, enhancing social support, increasing social contact, and addressing maladaptive social cognition [50]. The peer-based intervention in this study provided social opportunities for participants as well as informational, emotional and social support. Previous research has found that older people receiving telephone peer-based support services experienced significant improvements in self-confidence, independence, and social participation [21, 49]. In the present study, in-person visits were used to establish peer-based relationships with older people, due to the socio-cultural isolation faced by Chinese aging immigrants. This builds on previous research suggesting that face-to-face communication is a more effective way than digital communication to build peer support relationships and reduce loneliness [51]. Volunteers can see changes in older people during each visit and can instantly adjust the content or form of each conversation. This allows older people to feel that they have friends and can support re-engagement in their community, facilitating reduction of isolation and loneliness. The findings contribute to further validating the value of social impact theory in which shared age, ethnicity and language build the common grounds for the peers and the older people to connect with each other.

This study reveals a strong association between peer-based intervention and Chinese older immigrants’ improved resilience. Resilience, referring to the ability and resources to bounce back from adverse life conditions, has great benefits for older adults’ physical and mental health. The intervention provides older adults with emotional support and companionship, which is helpful to reduce loneliness, as well as informational support such as self-care skills and community resources that can support older adults’ longer-term recovery from adversities. Previous research has found that peer-based interventions can increase older adults’ resilience after stroke, which is beneficial for recovery [52]. Currently, peer-based interventions focus mainly on reducing loneliness in the short-term, and their effectiveness for long-term benefits, notably resilience, should receive more attention in future research.

In the current study, the treatment effects on psychosocial outcomes other than loneliness and resilience (e.g. barriers to social participation, anxiety, depression, social support, life satisfaction, purpose in life, happiness) were not significant. This indicates that the intervention was not as effective on other outcomes as for loneliness or resilience. In addition to the small sample size, another tentative explanation may be that the intervention is non-therapeutic and is rather brief. It may have greater impacts on loneliness, as it is experiential and based on resources acquired, compared to pathological (e.g. depression and anxiety) or long-term outcomes (e.g. happiness and life satisfaction) that may not be as easily altered. However, intervention group participants experienced an overall improvement in all psychosocial outcomes (except purpose in life), while no such changes were found in the control group. The significant within-group improvements indicate the potential effectiveness of this intervention on these outcomes, which calls for more studies in the future.

This study carries some limitations. First, the small sample size limits its generalizability, and most participants were over 80 years old. Future studies should include larger samples of Chinese older population from a wider age range. Second, as noted in the group assignment process in the methods section, a few special cases who showed obvious and urgent needs of social support were given priority to be included in the intervention group. While this approach would ethically address the urgent needs of the participants, this is also the reason for the mean age of the participants in the intervention group being older than the control group, thus risking the validity of the results being affected. Third, this study only involved one post-test after the intervention. In the future, more follow-up post-tests should be conducted to track the longer-term effects of the intervention. Future studies should examine the long-term effects of the peer-based interventions among the participants after the completion of the project. Fourth, only one peer intervention approach was tested, so its effectiveness in relation to other forms of intervention could not be fully established. Fifth, this study did not measure changes among the peer supporters, which could be a promising social activity for the young-old to contribute to society and achieve active aging, enabling them to better prepare for their own aging via interacting with older people. Future studies should measure psychosocial changes among peer supporters. Finally, this study did not adopt a standardized protocol. Repeating this type of intervention study is recommended so as to further address the potential problem of internal validity concerning whether the changes report were causally related to the intervention provided. While a more complicated research design to control for different intervention components may not be viable in real intervention context, development of explicit peer-support protocols and manual, and strengthening the systematic training for the peer-supporters would be useful to further ensure fidelity of the intervention.

However, this study has important implications for social work practice. On one hand, peer support is a critical supplement to professional services. The needs of immigrants and ethnic minority members can be complex, with the intersections of individual, cultural, and social contexts, which pose great demands and challenges for social workers. This compensation will become increasingly important as populations will inevitably become more diverse and multi-cultural [53, 54]. Comparing to services and support provided by professionals such as social workers and clinicians, peer support can be more effective as the former may have a risk of making the clients feel oppressed [55]. The use of peer-based interventions could be perceived as less stigmatizing and socially ‘natural’, thus attracting older people as well as their social support networks to participate in and benefit from interventions. This is particularly important for culturally diverse older adults who might feel uncomfortable using formal professional services [56, 57]. Mental illness has long been subject to public stigmatization and discrimination in Chinese culture, which makes some people reluctant to seek professional help [58]. Peer-based interventions can complement social work interventions in this regard, as peers share similar cultural background and experiences with service users. The peer-based intervention can help identify more older Chinese immigrants in social isolation but reluctant to seek professional help. In addition, the participants may disclose more unmet needs during the peer support process, which can be followed up and attended to by social workers and other health professionals even after the intervention ends. Moreover, there is a possibility that such peer-based interventions may be especially effective among Chinese populations, who may be deeply influenced by collectivist and interdependent social norms [59]. Moreover, peer-based interventions provided by neighbours and friends can be more affordable and continuous compared with professional social work services, and therefore can generate sustainable beneficial effects in an extended time period.

On the other hand, encouraging peer support can be helpful to promote the core values of social work practice, such as dignity, anti-oppressive practice, and strengths perspectives that are beneficial for both the support providers and receivers. It promotes empowerment human service agencies should adopt strategies to encourage more people in the community, especially the young-old, to volunteer as peers by promoting the idea of active aging. As future service users, such opportunities are beneficial for them to prepare and build stronger capacity for their own aging. Moreover, social work practitioners should maximize peer support in their professional social work practices by building interactional expertise and creating a dialogical space in group work contexts [55].

## Conclusion

The cost of providing care and support for the older people will increase, while a shortage of professionals is likely to affect practitioners’ capacity to attend to increasing demand within an increasingly diverse aging population. Peer-based interventions should be expanded, with protocols explored and refined to meet diversifying needs of older adults. Both short-term and long-term effectiveness should continue to be evaluated. Peer-based interventions can be beneficial for the health and well-being of both volunteers and support recipients. More importantly, they can empower the wider community by building community capacity wherein people support each other to address the challenges of elder care alongside formal service providers.

## Data Availability

The datasets used and/or analysed during the current study are available from the corresponding author on reasonable request.
